# Minimizing Selection and Classification Biases. Comment on “Clinical Characteristics and Prognostic Factors for Intensive Care Unit Admission of Patients With COVID-19: Retrospective Study Using Machine Learning and Natural Language Processing”

**DOI:** 10.2196/27142

**Published:** 2021-05-26

**Authors:** Francisco Martos Pérez, Ricardo Gomez Huelgas, María Dolores Martín Escalante, José Manuel Casas Rojo

**Affiliations:** 1 Department of Internal Medicine Hospital Costa del Sol Marbella Spain; 2 Internal Medicine Department Hospital Regional de Málaga Málaga Spain; 3 Internal Medicine Department Infanta Cristina University Hospital Parla (Madrid) Spain

**Keywords:** artificial intelligence, big data, COVID-19, electronic health records, tachypnea, SARS-CoV-2, predictive model, prognosis, classification bias, critical care

The paper by Izquierdo et al [1], published in the recent issue of the *Journal of Medical Internet Research*, employed a combination of conventional and machine learning tools to describe the clinical characteristics of patients with COVID-19 and the factors that predict intensive care unit (ICU) admission. We would like to make some comments about its design.

The authors should have provided the proportion of patients with a positive microbiological diagnosis. If the artificial intelligence software’s capacity for retrieving this information is limited in some way, this should be explained. The classification bias introduced by the lack of microbiological confirmation may have been significant since the study includes patients from January 1, 2020. Although some undiagnosed cases have likely been present prior to the first declared case (March 1, 2020) [2] in Castilla-La Mancha, it is improbable that there were many of them.

ICU admissions are related to many factors not addressed in the study. The decision not to admit a patient to the ICU because of short life expectancy, low quality of life, or high burden of comorbidities may have had a great impact during the first wave of the COVID-19 pandemic, when a scarcity of ICU beds manifested in some regions of Spain. The 6.1% ICU admission rate reported by the authors was 36% lower than the 8.3% reported in a national survey of 15,111 patients from 150 hospitals in Spain [3]. We could hypothesize that the patients included in the study had a milder form of the disease. However, given the absence of a microbiological diagnosis in an unknown percentage of patients, the inclusion of a significant proportion of patients without a real COVID-19 diagnosis cannot be ruled out. These doubts could have been resolved if a microbiological diagnosis had been a requisite for inclusion. The mortality rate, the most robust and relevant endpoint, should also have been reported and the factors related to it analyzed.

Artificial intelligence is having an increasing impact on the rate of health care information processing. However, minimization of selection and classification biases should be guaranteed in the design of investigations. In this case, this could have been achieved by including only microbiologically confirmed cases and prolonging the period of inclusion, since most COVID-19 cases emerged after the end date of the study inclusion period. These changes in the design would have allowed for a better evaluation of the performance of artificial intelligence techniques, making the results obtained in the sample closer to those of the real population.

## Abbreviations

ICU: intensive care unit

## References

[1] Izquierdo Jose Luis, Ancochea Julio, Soriano Joan B, Savana COVID-19 Research Group (2020). Clinical Characteristics and Prognostic Factors for Intensive Care Unit Admission of Patients With COVID-19: Retrospective Study Using Machine Learning and Natural Language Processing. J Med Internet Res.

[2] (2020). Europa Press.

[3] Casas-Rojo JM, Antón-Santos  JM, Millán-Núñez-Cortés J, Lumbreras-Bermejo C, Ramos-Rincón JM, Roy-Vallejo E, Artero-Mora A, Arnalich-Fernández F, García-Bruñén J M, Vargas-Núñez JA, Freire-Castro SJ, Manzano-Espinosa L, Perales-Fraile I, Crestelo-Viéitez A, Puchades-Gimeno F, Rodilla-Sala E, Solís-Marquínez M N, Bonet-Tur D, Fidalgo-Moreno M P, Fonseca-Aizpuru E M, Carrasco-Sánchez F J, Rabadán-Pejenaute E, Rubio-Rivas M, Torres-Peña J D, Gómez-Huelgas R (2020). Clinical characteristics of patients hospitalized with COVID-19 in Spain: Results from the SEMI-COVID-19 Registry. Rev Clin Esp (Barc).

